# Development and optimization of orthotopic liver metastasis xenograft mouse models in uveal melanoma

**DOI:** 10.1186/s12967-020-02377-x

**Published:** 2020-05-20

**Authors:** Takahito Sugase, Bao Q. Lam, Meggie Danielson, Mizue Terai, Andrew E. Aplin, J. Silvio Gutkind, Takami Sato

**Affiliations:** 1 Department of Medical Oncology, Sidney Kimmel Cancer Center, Thomas Jefferson University, 1015 Walnut Street, Ste. 1024, Philadelphia, PA USA; 2 Department of Cancer Biology, Sidney Kimmel Cancer Center, Thomas Jefferson University, Philadelphia, PA USA; 3 Department of Pharmacology, Moores Cancer Center, University of California San Diego, La Jolla, CA 92093 USA

**Keywords:** Uveal melanoma, Orthotopic xenograft model, Liver metastasis, Spleen, Liver

## Abstract

**Background:**

Patients with metastatic uveal melanoma (MUM) in the liver usually die within 1 year. The development of new treatments for MUM has been limited by the lack of diverse MUM cell lines and appropriate animal models. We previously reported that orthotopic xenograft mouse models established by direct injection of MUM cells into the liver were useful for the analysis associated with tumor microenvironment in the liver. However, considering that patients with UM metastasize to the liver hematogenously, direct liver injection model might not be suitable for investigation on various mechanisms of liver metastasis. Here, we aim to establish new orthotopic xenograft models via hematogenous dissemination of tumor cells to the liver, and to compare their characteristics with the hepatic injection model. We also determine if hepatic tumors could be effectively monitored with non-invasive live imaging.

**Methods:**

tdtTomate-labeled, patient-derived MUM cells were injected into the liver, spleen or tail vein of immunodeficient NSG mice. Tumor growth was serially assessed with In Vivo Imaging System (IVIS) images once every week. Established hepatic tumors were evaluated with CT scan and then analyzed histologically.

**Results:**

We found that splenic injection could consistently establish hepatic tumors. Non-invasive imaging showed that the splenic injection model had more consistent and stronger fluorescent intensity compared to the hepatic injection model. There were no significant differences in tumor growth between splenic injection with splenectomy and without splenectomy. The splenic injection established hepatic tumors diffusely throughout the liver, while the hepatic injection of tumor cells established a single localized tumor. Long-term monitoring of tumor development showed that tumor growth, tumor distribution in the liver, and overall survival depended on the number of tumor cells injected to the spleen.

**Conclusion:**

We established a new orthotopic hepatic metastatic xenograft mouse model by splenic injection of MUM cells. The growth of orthotopic hepatic tumors could be monitored with non-invasive IVIS imaging. Moreover, we evaluated the therapeutic effect of a MEK inhibitor by using this model. Our findings suggest that our new orthotopic liver metastatic mouse model may be useful for preclinical drug screening experiments and for the analysis of liver metastasis mechanisms.

## Background

Uveal melanoma (UM), which originates from melanocytes within the iris, choroid, and ciliary body, is a rare disease but the most frequent non-cutaneous melanoma and the most frequent primary cancer of the eye in adults [1, 2]. Up to 50% of patients with primary UM develop metastases, typically in the liver via the hematogenous route within 15 years of initial diagnosis with a peak of metastasis between 2 and 5 years [2, 3]. The median survival after diagnosis of metastatic UM (MUM) is approximately 1 year [4, 5]. Currently, there are no U.S. Food and Drug Administration (FDA)-approved therapies for MUM [6], and overall survival among individuals diagnosed with MUM has not significantly changed between 1973 and 2009 [1, 7–10].

To develop new therapeutic strategies, in vitro and preclinical models of MUM are critical; however, only a few MUM cell lines and preclinical mouse models are available for research. Many researchers have used either a subcutaneous injection of cell lines derived from primary UM or retro-orbital injection of liver-selected murine cutaneous melanoma B16 cells [11–13]. Subcutaneous heterotopic mouse models are commonly used in cancer research because this model does not require labor-intensive or technically demanding procedures. However, the genetics of UM contrast with that of cutaneous melanoma [1, 14] and therapeutic regimens that have demonstrated promising results in the subcutaneous heterotopic mouse model often have little effect on cancer patients [15, 16]. Thus, the development of more biologically relevant animal models to test therapeutic strategies in advanced-stage UM is required.

The orthotopic xenograft mouse model is believed to resemble natural tumorigenesis in humans because this model has a similar tumor microenvironment of the original tumor [17]. We have previously reported that TJU-UM001 cell line, which was established from liver metastasis of UM patients in our laboratory, could establish orthotopic hepatic tumors in the mouse liver, but showed no success in developing a tumor by subcutaneous injection. This result indicates that the mouse liver is a suitable microenvironment to support the development of MUM tumors [18]. Moreover, we investigated the potential resistant mechanisms to medications by using our orthotopic liver metastatic mouse model. The association between hepatic MUM tumors and several molecules secreted from hepatic stellate cells (HSCs) [16, 19, 20] was identified. Chua V et al. revealed that fibroblast growth factor 2 (FGF2), which is secreted from HSCs, rescued MUM cells from growth inhibition by BET inhibitors. They demonstrated that orthotopic liver metastatic tumors in the presence of FGF2 were ineffective with BET inhibitor, and the combination of FGFR inhibitor and BET inhibitor significantly suppressed tumor growth in the liver microenvironment [16].

Considering that UM tends to metastasize to the liver hematogenously, direct liver implantation model might not be suitable for investigation on mechanism of liver metastasis; therefore, the establishment of a new orthotopic liver metastatic mouse model via hematogenous dissemination is required. Here, we hypothesized that MUM cells injected into the spleen or tail vein would disseminate into the liver from the spleen via the splenic vein and portal vein, or from tail vein via systemic circulation through the heart. In this study, we performed splenic injection and tail vein injection to establish a new orthotopic liver metastatic model and then compared the results with the hepatic tumors in our previous mouse models. Finally, we investigated whether hepatic tumors could be monitored with non-invasive live imaging.

## Materials and methods

### Cell lines

**Fig. 1:**
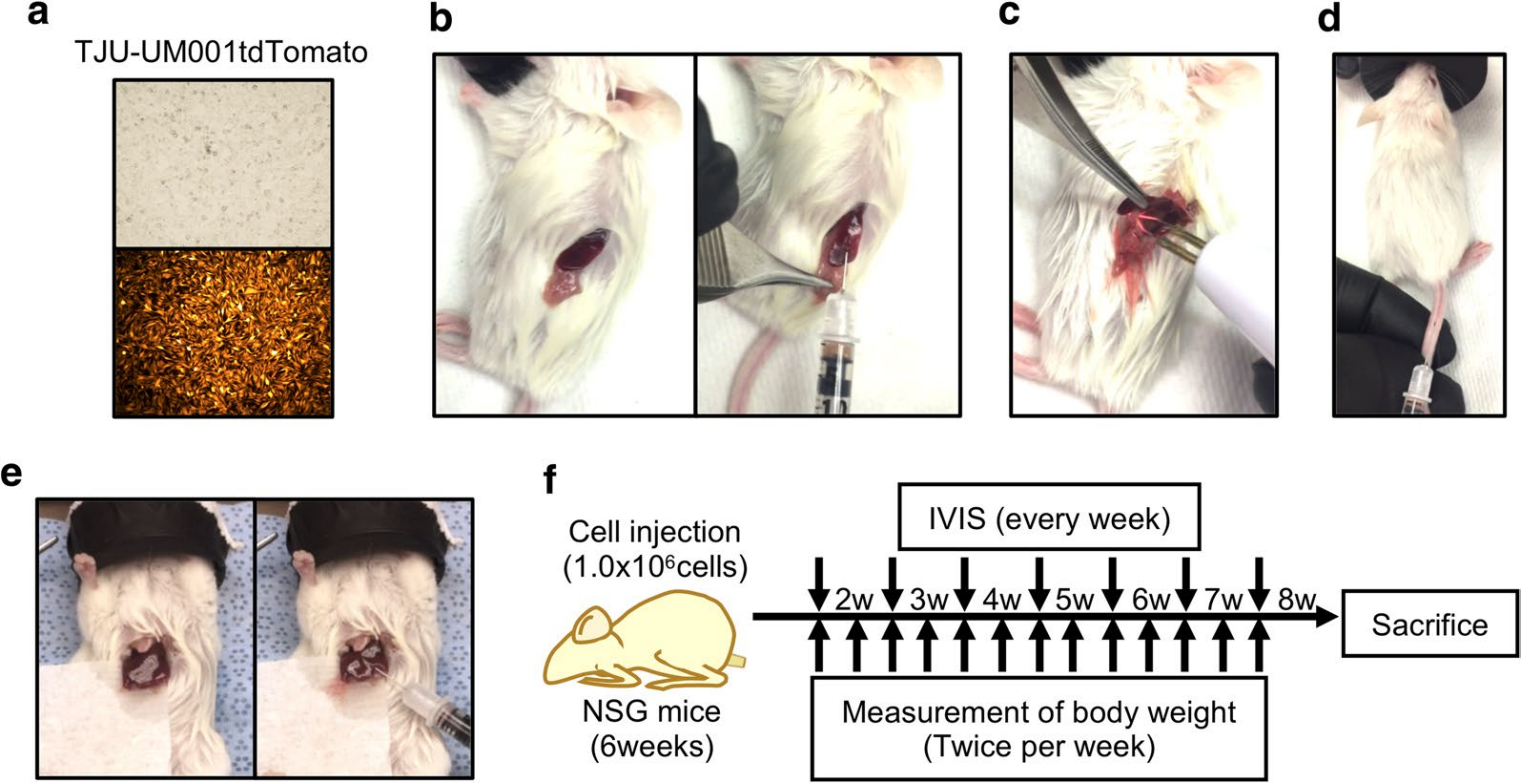
**a** TJU-UM001-tdTomato cells express orange-red fluorescence (× 100). NSG mouse (6- to 8-weeks old) were injected with tumor cells (1.0x10^6^ cells/mouse) into the spleen, the liver, or the tail vein. Surgical procedure **b** splenic injection, **c** splenectomy, **d** tail vein injection, and **e** hepatic injection. **f** Monitoring schedule

TJU-UM001 and TJU-UM004 cell lines were established in our laboratory at Thomas Jefferson University and authenticated by DDC Medical (Fairfield, OH, USA). They are derived from a liver metastasis and an orbital metastasis of human UM, respectively. Both UM001 and UM004 cells harbor the Q209P mutation as determined by Sanger DNA sequencing as previously described [21]. UM001-tdTomato cells were established as previously described [22], and cultured in RPMI 1640 supplemented with 10% FBS, 10% non-essential amino acids, 2 mM l-glutamine, 10 mM HEPES buffer, 50 IU/ml penicillin, and 50 mg/ml streptomycin (Fig. 1a). UM004 cells were cultured in MEM medium containing 10% FBS, 50 IU/ml penicillin and 50 mg/ml streptomycin. All cell lines were maintained in a humidified atmosphere (5% CO2) at 37 °C.

### Mouse

NOD.Cg-Prkdc^scid^ Il2rg^tm1Wjl^/SzJ (NSG) mice were purchased from The Jackson Laboratory (Bar Harbor, ME), and bred and kept in filter top cages at 22 ^°C^, 60% humidity in our facility. Both male and female 8- to 10-week-old mice received surgery and were kept under the same conditions.

### Surgery

Mice were placed on heating pad and anesthetized with 3% isoflurane for induction and 2% for maintenance. 70% ethanol was sprayed on the abdomen or tail before surgery or injection. Postoperatively, mice were kept warm with a heater and returned to their cages when fully awake. The animal study was approved by the Institutional Animal Care and Use Committee of Thomas Jefferson University and adhered to the recommendations in the National Institutes of Health Guide for the Care and Use of Laboratory Animals.

### Splenic injection model

Mice were placed in the right lateral recumbent position. A 1-cm incision was made in the left upper abdominal wall, followed by a 1-cm incision in the peritoneum to expose the spleen. 0.25–2.0 × 10^6^ cells in 20 μl of RPMI 1640 were gently injected into the spleen (Fig. 1b). The insertion site of the needle was cauterized and sealed with absorbable hemostatic material (SURGICEL, Johnson and Johnson, New Brunswick, NJ, USA) to curtail bleeding. Splenectomy was performed 15 min after injection using surgical cautery tip (#231; McKesson, San Francisco, CA) (Fig. 1c). The abdominal incision was closed in two layers with 5-0 polydioxanone absorbable thread (AD Surgical, Sunnyvale, CA, USA).

### Tail vein injection model

Mice were placed in the prone position. 1.0 × 10^6^ UM001-tdTomato cells in 20 μl of RPMI 1640 were gently injected into tail vein (Fig. 1d). After injection, gentle pressure was applied with fingers until any bleeding stopped.

### Hepatic injection model

Mice were placed in the supine position. A 1- to 1.5-cm skin incision was made in the upper abdominal wall, followed by a 1-cm incision in the peritoneum to expose the liver. The left lobe of the liver was moved outside the body and placed on a nonwoven absorbent fabric sheet as previously described [18]. 1.0 × 10^6^ UM001-tdTomato cells in 20 μl of 2:1 RPMI 1640/Matrigel (BD Biosciences, Bedford, MA, USA) were gently injected under the surface of the left lobe of the liver (Fig. 1e). The insertion site of the needle was cauterized and sealed with absorbable hemostatic material. The liver was returned within the body after the injection, and the abdominal incision was closed in 2 layers with 5-0 polydioxanone absorbable thread (AD Surgical, Sunnyvale, CA, USA).

### In vivo imaging

Mice were anesthetized by the XGI-8 Gas Anesthesia system (2% isoflurane; Xenogen, Alameda, CA, USA). After shaving the fur, fluorescent intensity was measured by In Vivo Imaging System (IVIS) Lumina XR (Xenogen). To acquire an image sequence, Living Image Ver.4.5 (Xenogen) image software was used. The region of interest was drawn in the upper abdominal area, and the photon flux data was measured.

### CT scan

Micro-CT scan (Inveon Micro-CT, Siemens, Germany) was performed 1 day after injection of a contrast agent (ExiTron nano 12000, Miltenyi Biotec, Germany) which is an alkaline earth-based nanoparticulate contrast agent for mouse liver CT imaging [23]. Mice were injected with 100 μl of contrast agent via a lateral tail vein. Since this agent is taken up by cells of the reticuloendothelial system including macrophages within the liver and spleen, normal liver and spleen were enhanced and tumors were drawn as a black spot.

### Histology and immunohistochemistry

For immunohistochemistry, 5 μm tissue sections were steamed for 20 min with antigen retrieval solution and stained with primary antibodies, SOX10 (A-2, sc-365692; Santa Cruz Biotechnology, Santa Cruz, CA), HMB45 (M0634; Dako, Carpentaria, CA) and S100 (Z0311; Dako, Carpentaria, CA) overnight at 4 °C. On the next day, sections were incubated for 30 min in Imm-PRESS AP Reagent (Vector Laboratories, Burlingame, CA, USA), followed by 5 min incubation with ImmPACT NOVA-RED (Vector Laboratories).

### Therapeutic intervention on UM hepatic metastasis

We evaluated the therapeutic effects of a MEK inhibitor (trametinib) on UM liver metastasis using the splenic injection model. 6 weeks after UM001tdTomato cell injection into the spleen, mice were treated with control (dilute solution) or MEK inhibitor, trametinib (1.0 mg/kg) intraperitoneally once a day for 3 weeks. Each tumor growth was monitored by IVIS imaging once a week and body weights were measured twice a week after cell injection. All mice were sacrificed after each treatment.

### Statistical analysis

Data of xenograft mouse models were shown as means ± standard errors of the means (SEMs). Unpaired Student’s t-tests were used to test for statistically significant differences between 2 groups; 2-sided P values less than 0.05 were considered significant. Overall survival was evaluated using the Kaplan–Meier method, and differences were compared using the log-rank test. These analyses were carried out using JMP Pro version 13.0 (SAS Institute, Cary, NC).

## Results

### Establishment of various orthotopic xenograft models which were injected into the spleen, liver, or tail vein

NSG mice were injected with UM001-tdTomato cells (1.0x10^6^ cells/mouse) (Fig. 1a) into the spleen, tail vein, or liver. We investigated the establishment of hepatic tumors in splenic injection with splenectomy (Fig. 1b, c), splenic injection without splenectomy, or tail vein injection (Fig. 1d). We compared these mouse models with the hepatic injection model (Fig. 1e). IVIS images were taken every week and their body weights were measured twice a week starting at 2 weeks after injection and continuing up to 8 weeks after injection (Fig. 1f).

**Fig. 2:**
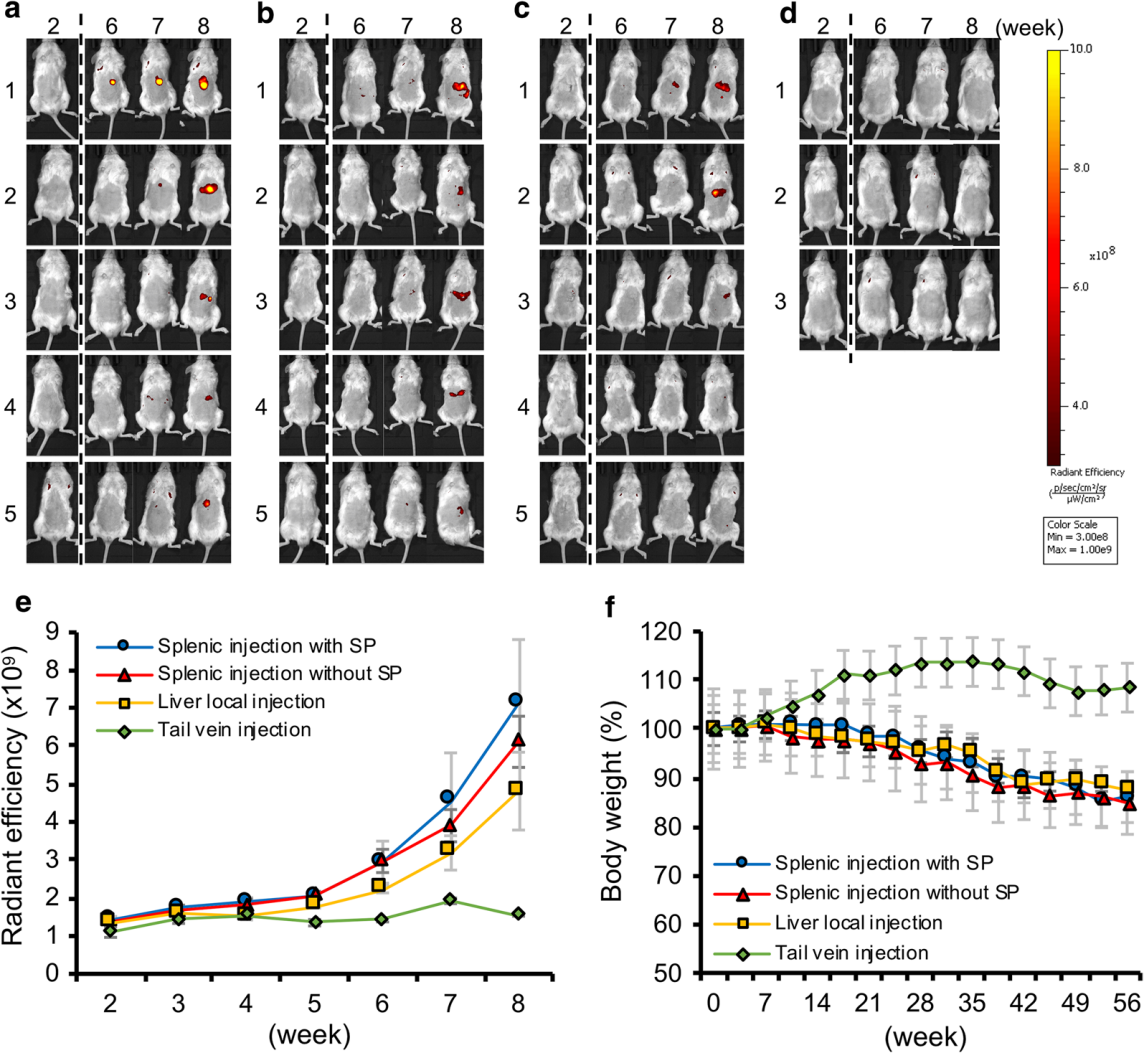
Each mouse model was taken IVIS image every week. IVIS images at 2, 6, 7 and 8 weeks after injection were shown: **a** splenic injection with splenectomy (SP), **b** splenic injection without SP, **c** hepatic injection, and **d** tail vein injection. **e** Weekly measurement of fluorescent intensity. Data were shown with the mean radiant efficiency ± SEMs of 5 mice in each cohort. **f** Shift of mouse body weights. Values were shown with the mean ± SEMs

All mice with MUM cells injected into the spleen showed strong fluorescent signals in the upper abdomen at 8 weeks after injection (Fig. 2a, b). One mouse with splenic injection without splenectomy showed detectable fluorescent signals in the left flank in addition to the upper abdomen (Fig. 2b1). On the other hand, 3 out of 5 hepatic injection mice also showed strong fluorescent signals in the upper abdomen, but the other 2 mice showed relatively weak or no detectable fluorescent signals (Fig. 2c). Meanwhile, all mice with MUM cells injected into the tail vein showed no detectable fluorescent signals (Fig. 2d). Figure 2e shows the summary of fluorescent intensity in each mouse group. The fluorescent intensity of splenic injection group and hepatic injection group increased in a time-dependent manner, but the fluorescent intensity of the splenic injection group was relatively higher compared to the liver injection group. No increase of fluorescent intensity was observed in the tail vein injection group (Fig. 2e). The splenic injection group and hepatic injection group experienced a consistent decrease in body weight, while no loss of weight was observed in the tail vein injection group (Fig. 2f).

### Comparison of hepatic tumors in the spleen-, liver-, and tail vein-injected orthotopic xenograft models

**Fig. 3:**
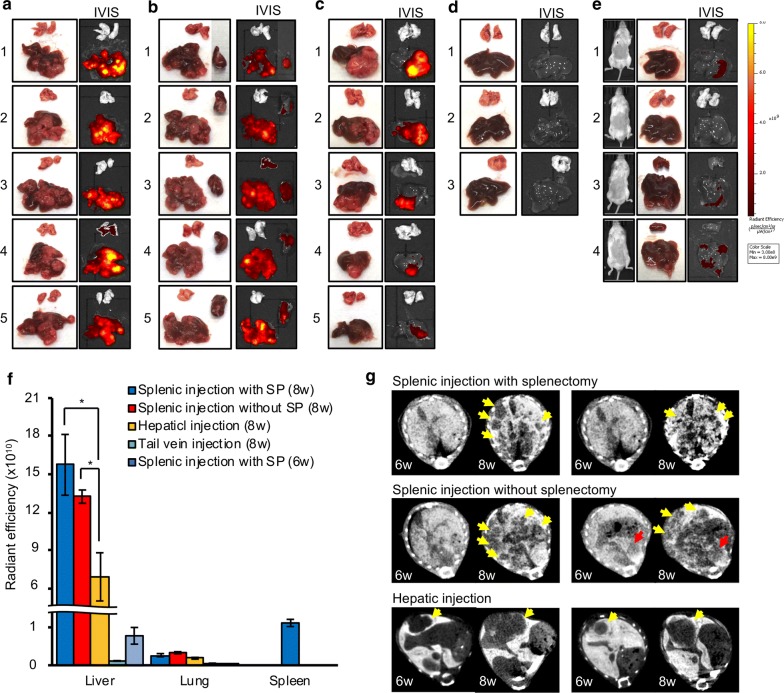
Ex vivo image and IVIS image of removed liver, lung, and spleen at 8 weeks after injection: **a** splenic injection with splenectomy, **b** splenic injection without splenectomy, **c** hepatic injection, and **d** tail vein injection. **e** Live IVIS images (left) and IVIS images of extracted liver and lung (right) at 6 weeks after splenic injection with splenectomy. **f** Fluorescent intensity of liver, lung and spleen in each mouse model. Data were the mean radiant efficiency ± SEMs of 5 mice in each mouse model. **g** CT scan images of tumors in 6 weeks and 8 weeks after injections. Yellow arrows: tumor in the liver. Red arrows: tumor in the spleen

At the conclusion of above experiments, all mice were sacrificed and the liver and lung were removed. IVIS images of the liver and lungs in each mouse group were taken immediately after removal of individual organs. In the splenic injection model, multiple hepatic tumors were established throughout the liver (Fig. 3a, b), while hepatic tumors of the hepatic injection model were established only in the left lobe in which cells were injected (Fig. 3c). Some mice with splenic injection showed detectable fluorescent signals even in the lungs (Fig. 3a4 and b3). All splenic injection mice without splenectomy showed detectable fluorescent signals in the spleen (Fig. 3b). Meanwhile, in the tail vein injection model, no detectable fluorescent signals were observed in the liver and lung (Fig. 3d). Additionally, in the splenic injection mice with splenectomy, IVIS images taken 6 weeks after the injection revealed no detectable fluorescent signals in vivo, but weaker fluorescent signals could be detected in the liver ex vivo (Fig. 3e).

We summarized the fluorescent intensity of the liver, lung, and spleen ex vivo in each mouse model. There were no significant differences of fluorescent intensity in the liver between splenic injection models with splenectomy and without splenectomy. Hepatic injection model showed significantly weaker fluorescent intensity in the liver compared to splenic injection model (splenectomy; P = 0.038 and non-splenectomy; P = 0.047, respectively). The splenic injection model with splenectomy showed slightly increased fluorescence even after 6 weeks, however fluorescent readings of the tail vein injection model after 8 weeks showed undetectable changes. Regarding the lung involvement in these models, the splenic injection model and hepatic injection model showed a slight increase of fluorescent intensity at 8 weeks after injection. In the splenic injection model without splenectomy, the spleen also showed strong fluorescent intensity (Fig. 3f).

### CT image of splenic injection model and hepatic injection model

In the splenic injection model, no hepatic tumors could be detected at 6 weeks after injection, but multiple hepatic tumors (yellow arrow) were observed at 8 weeks after injection. Tumors were observed at 6 weeks after injection in the spleen (red arrow). In hepatic injection model, a single hepatic tumor (yellow arrow) was observed at 6 weeks after injection and progressed 2 weeks later (Fig. 3g).

### Immunohistochemistry (IHC) analysis in each mouse model

**Fig. 4:**
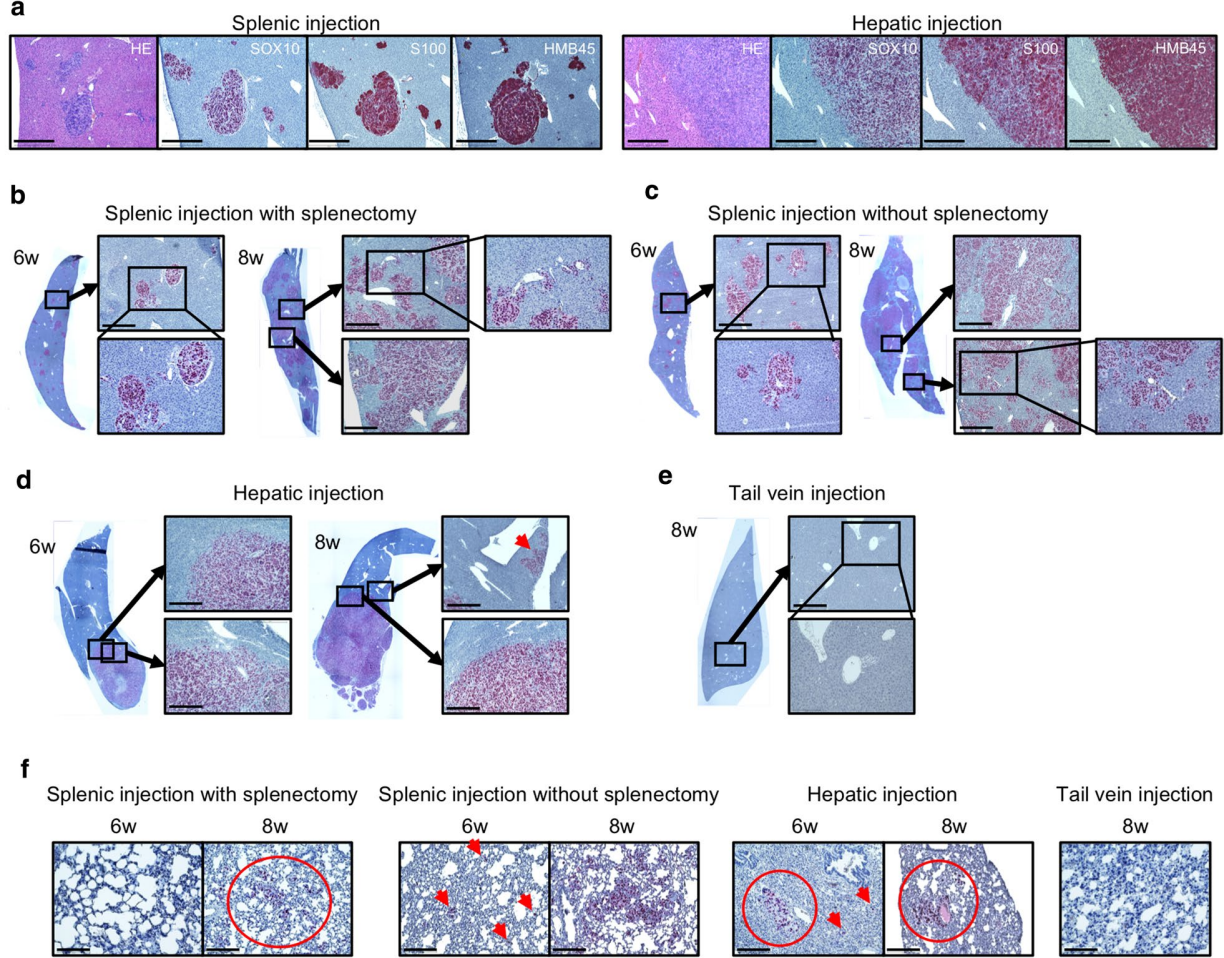
Immunohistochemical analysis of hepatic tumor. **a** Comparison of SOX10, S100 and HMB45 expression in the liver of splenic injection model and hepatic injection model. Liver tissues in each mouse model at 6 and 8 weeks after injection were stained with SOX10 (100x). Liver 6 weeks or 8 weeks after injection of tumor cells; **b** splenic injection with splenectomy, **c** splenic injection without splenectomy, **d** hepatic injection and **e** tail vein injection (100 × and 200x). **f** Lung in each model were stained with SOX10 (100x). Scale bar = 500 μm

SOX10 is a neural crest transcription factor that plays an important role in the specification of Schwann cells and melanocytes [24, 25] and has recently proved to be the most sensitive marker for UM [26–28]. Therefore, we stained orthotopic hepatic tumors with SOX10 in addition to S100 and HMB45, which have been used as specific antibodies to UM. SOX10 was expressed in hepatic tumors established by splenic injection as well as by hepatic injection, as similar to S100 and HMB45 (Fig. 4a). As a next step, we performed IHC staining at 6 and 8 weeks after injection in each mouse. In the splenic injection model, multiple small tumor colonies were observed near the blood vessels at 6 weeks after injection. These tumor colonies had grown to occupy the majority of the liver by two weeks later (8 weeks after injection) (Fig. 4b, c). In the hepatic injection model, the injected cells settled at the injection site, and a single localized tumor was observed at 6 weeks after injection. This tumor gradually grew locally and metastasized throughout the liver via intrahepatic vessels (red arrow) at 8 weeks after injection (Fig. 4d). In the tail vein injection model, there were no SOX10 positive cells in the liver (Fig. 4e).

We performed SOX10 staining of lung samples to confirm lung metastasis in each mouse. Lung metastases were observed in the splenic injection model with splenectomy at 8 weeks after injection but not at 6 weeks after injection. In the splenic injection model without splenectomy and in the hepatic injection model, a few SOX10-positive cells were observed at 6 weeks after injection and 2 weeks later the lung metastasis had progressed. No SOX10 positive cells were observed in the lung of the tail vein injection model (Fig. 4f).

### Long-term monitoring in splenic injection with splenectomy model

We confirmed that the orthotopic xenograft mouse model of liver MUM could be established by splenic injection with splenectomy in the aforementioned animal experiments. We next performed long-term monitoring of the hepatic tumor with IVIS imaging in this model. NSG mice were injected with UM001-tdTomato cells (2.0, 1.0, 0.5 and 0.25x10^6^ cells/mouse, 5 mice of each group) into the spleen, and then the splenectomy was performed 15 min after injection. Weekly IVIS images were taken from each mouse until 16 weeks after injection. If mice show 20% weight loss or severe weakness, they will be euthanized using C0_2_ exposure followed by cervical dislocation.

**Fig. 5:**
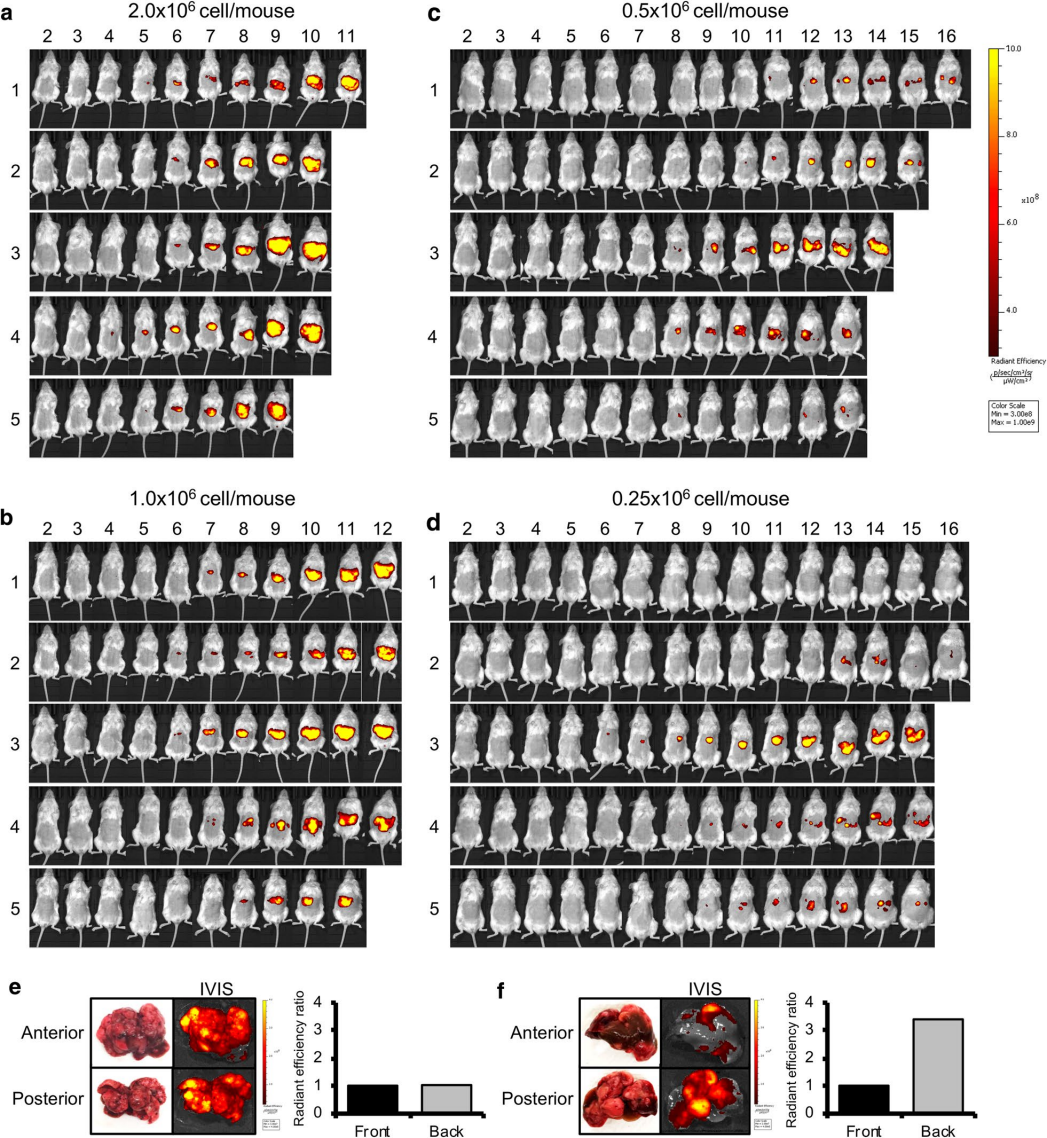
Long term monitoring of hepatic tumor with IVIS image was performed in splenic injection with splenectomy model. NSG mice were injected UM001-tdTomato cells (2.0, 1.0, 0.5, and 0.25x10^6^ cells/mouse, 5 mice of each group) into spleen, then spleen was removed 15 min after injection. IVIS image has been taken every week and mice will be euthanized using C0_2_ exposure followed by cervical dislocation if they show 20% weight loss or severe weakness. **a** 2.0x10^6^ cells/mouse, **b** 1.0x10^6^ cells/mouse, **c** 0.5x10^6^ cells/mouse and **d** 0.25x10^6^ cells/mouse. The fluorescent intensity between the anterior portion and the posterior portion of the liver in the **e** 1.0 and **f** 0.25x10^6^ cells injected mice were compared

All mice with 2.0x10^6^ cells injection showed detectable fluorescent signals at 6 weeks after injection. Hepatic tumors aggressively progressed, and strong fluorescent signals were detected throughout the upper abdomen at 9–10 weeks (Fig. 5a). All mice with 1.0x10^6^ cells injection showed detectable fluorescent signals at 8 weeks after injection. The growth of hepatic tumors was relatively slow at the beginning compared to mice with 2.0x10^6^ cells injection; however, strong fluorescent signals became detectable throughout the upper abdomen at 11–12 weeks after injection. The intensity of fluorescent signals was similar to those of mice with 2x10^6^ cells injection (Fig. 5b). In mice with 0.5 and 0.25x10^6^ cells injection, one of 5 mice in each group showed a continuous increase in fluorescent intensity that was similar to mice with 2.0 and 1.0x10^6^ injection (Fig. 5c3 and d3), but their growth was very slow. Most of the other mice showed detectable fluorescent signals in a part of the upper abdomen, but their positive signals were unevenly distributed (Fig. 5d). All mice with less than 0.5x10^6^ cells injection also developed diffuse hepatic tumors, and one mouse with 0.25x10^6^ cells injection survived over 16 weeks after injection (Fig. 5d1).

We next compared the distribution of tumors in the liver between mice with 1.0 and 0.25x10^6^ cells injection. In mice with 1.0x10^6^ cell injection, tumors were evenly distributed in the liver, and there were no differences of in fluorescent intensity between the anterior portion and the posterior portion of the liver at 12 weeks after splenic injection (Fig. 5e). A liver sample with 0.25x10^6^ cell injection was obtained immediately after death at 16 weeks after injection (Fig. 5d2). This mouse had multiple small masses of hepatic tumors unevenly distributed in the liver, and large differences of fluorescent intensity were observed between the anterior portion and the posterior portion of the liver (Fig. 5f).

**Fig. 6:**
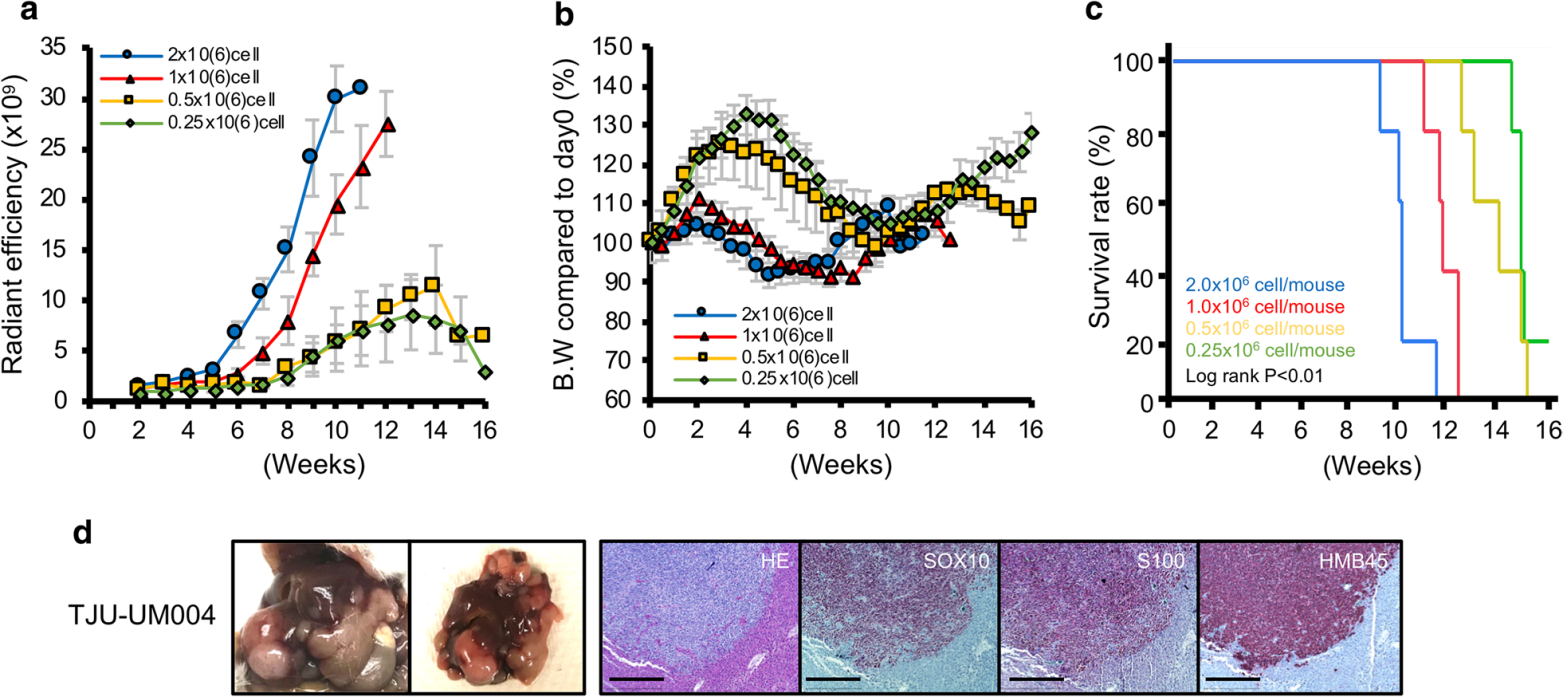
**a** Fluorescent intensity in the splenic injection with splenectomy was monitored every week. Data were the mean radiant efficiency ± SEMs of 5 mice in each mouse model. **b** Mice body weights were measured twice per week. Values shown represent the mean ± SEMs. **c** Survival curve based on the number of injected cells (5 mice in each mouse model). Survivals were compared using the log rank test. **d** NSG mouse was injected with TJU-UM004 cells (1.0x10^6^ cells/mouse)into the spleen and sacrificed 9 weeks after injection. HE staining and immunohistochemistry of SOX10, S100, and HMB45 were performed (100x). Scale bar = 500 μm

We summarized fluorescent intensity of each mouse model cohort in Fig. 6a. The fluorescent intensity of mice with 2.0x10^6^ cell injection increased at 5–6 weeks after injection. Mice with 1.0x10^6^ cell injection also showed similar tumor growth curve with a 1–2 week delay. In mice with less than 0.5x10^6^ cells injection, fluorescent intensity did not show the similar growth curves as seen in mice with 1.0x10^6^ cell injection (Fig. 6a). All mice tended to experience a loss in body weight when hepatic tumors were established, and then their weight increased as the hepatic tumors became larger. This tendency was observed earlier as the number of injected cells increased (Fig. 6b). Survival after splenic injection was associated with the number of injected cells (overall survival [median (range)]; 2.0, 1.0, 0.5 and 0.25x10^6^ cells cohorts, 73.6 (66–84) days, 86.6 (80–91) days, 103.2 (92–113) and 110.5 (108–112) days, respectively), and these differences were significant (Log rank P < 0.01) (Fig. 6c).

### Establishment of orthotopic hepatic tumor by injection of another UM cell line into the spleen

We injected another MUM cell line into the spleen to investigate whether other types of hepatic tumor models could be established. Splenic injection of UM004 cells, which are established from UM orbital metastasis, established hepatic tumors that were similar to the UM001 cells. These tumors also expressed SOX10, S100, and HMB45 similar to hepatic tumors established from UM001 (Fig. 6d).

### Evaluation of therapeutic effect on UM liver metastasis using spleen injection model

**Fig. 7:**
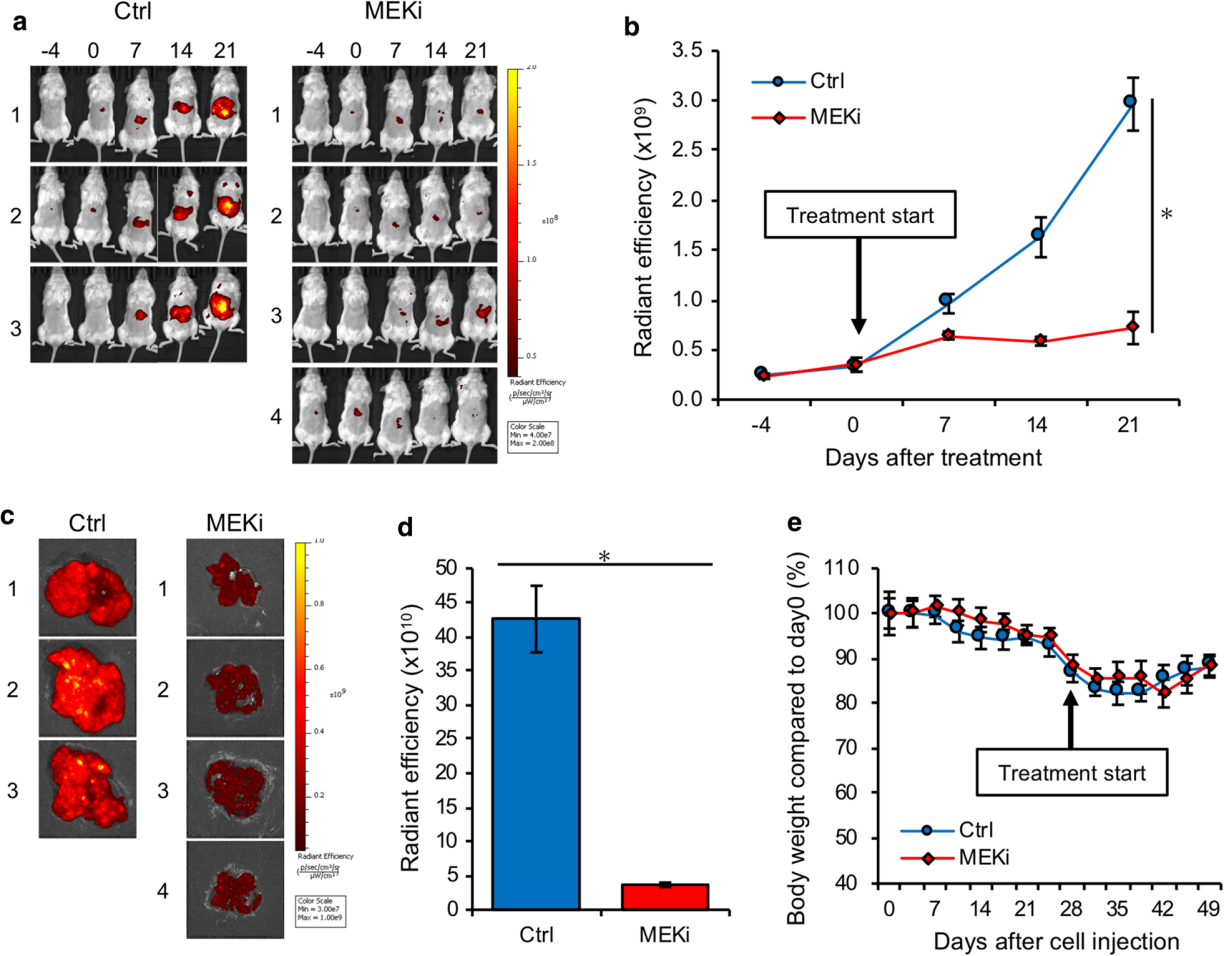
6 weeks after UM001tdTomato cell injection into the spleen, mice were treated with control (dilute solution) or MEK inhibitor, trametinib, (1.0 mg/kg) intraperitoneally once a day for 3 weeks. **a** IVIS images in each mouse were shown. **b** The measurement of fluorescent intensity. Data were shown with the mean radiant efficiency ± SEM in each cohort. **c** IVIS image of removed liver at 21 days after treatment. **d** Fluorescent intensity of liver in each cohort. Data were the mean radiant efficiency ± SEMs in each cohort. **e** Mice body weight were measured twice per week in each cohort. Values were shown with the mean ± SEMs

Mice treated with a MEK inhibitor, trametinib, showed weaker fluorescent signals than those treated with control (Fig. 7a), and the MEK inhibitor significantly suppressed hepatic tumor growth compared with that in mice treated with control (Fig. 7b). The removed liver in the both groups showed that the injected cancer cells have disseminated throughout the liver, but significantly weaker fluorescent signals were detected in the liver treated with the MEK inhibitor (Fig. 7c, d). There were no significant differences of body weight between both groups (Fig. 7e).

## Discussion

Recently, various orthotopic xenograft mouse models focusing on tumor microenvironment have been established using cancer cell lines or human cancer specimens as clinically relevant cancer models. Most of them have been established by direct injection or implantation into the original organs with a high success rate [29–34]. We previously reported that orthotopic xenograft mouse models established by direct injection of MUM cells into the liver were useful for the analysis associated with tumor microenvironment [16, 18–20, 22]. However, this direct hepatic injection model has some technical challenges and limitations related to the surgical procedure. First, slight leakage of tumor cell suspensions was frequently observed even if the cell suspension mixed with matrigel was carefully injected into the liver. Therefore, the growth of hepatic tumor in these mice was inconsistent (Figs. 2c and 3c). Second, the location of the tumors in the liver was slightly different in each mouse due to the depth and angle of the needle at the time of injection. As shown in the CT images, the hepatic tumors at the deeper sites of the liver were surrounded by normal tissue and as such, IVIS assessment may underestimate these tumors compared to tumors exposed on the liver surface.

In order to overcome these problems, new orthotopic mouse models mimicking liver metastasis of UM are needed. In our current study, we demonstrate that splenic injection of MUM cell lines can establish orthotopic hepatic tumors throughout the liver due to hematogenous dissemination. Surprisingly, the splenic injection model had little leakage compared to hepatic injection model even if matrigel-free cells were injected into the spleen. Moreover, since this approach disseminates most of the injected cells immediately throughout the liver via the splenic and portal veins, the splenic injection model resulted in less disparity in the number and the location of disseminated tumor cells compared to the hepatic injection model. Our present study showed that the mice that received the splenic injection established hepatic tumors with similar histological characteristics compared to those established by intra-hepatic injection.

To monitor the hepatic tumor in each mouse model, we used IVIS live imaging and CT scans. In the splenic injection model, IVIS imaging could monitor diffusely-distributed tiny hepatic tumors which are undetectable by CT scan (Figs. 2 and 3). IVIS imaging is considered to be more suitable than CT scan for monitoring hepatic tumors in splenic injection model since it can be difficult to identify the boundaries of multiple small tumors in the liver and monitor each one of hepatic tumors. On the other hand, CT scan can measure the size of tumors accurately regardless of the tumor location. Since the hepatic injection model can identify a single hepatic tumor with CT scan, CT scan is considered to be more suitable for use in animal experiments with the hepatic injection model, as has been previously reported [16]. Thus, selecting the appropriate monitoring of tumor size and volume is required depending on each injection model.

Short-term monitoring with IVIS imaging revealed no differences in established hepatic tumors between the splenectomy and non-splenectomy groups. These results suggested that most injected cells disseminated to the liver immediately after splenic injection and that the cancer cells colonized in the spleen have little effect on the establishment of liver metastasis. Since tumors in the spleen potentially influence the assessment of hepatic tumor with IVIS imaging (Fig. 2b1), we considered that the splenic injection with splenectomy model to should be more suitable for future preclinical experiments. In order to investigate the growth of hepatic tumors and survival in this model, we performed long-term monitoring with IVIS imaging in mice that were injected with different numbers of cells. In this present study, orthotopic hepatic tumors developed in all mice regardless of the number of injected cells, and survival was associated with the number of injected cells. IVIS monitoring revealed that all mice that were injected with more than 1.0 × 10^6^ cells into the spleen showed continuous growth of hepatic tumor. On the other hand, in mice with less than 0.5x10^6^ cells injection, the fluorescent intensity was sporadic and variable despite the fact that the tumor was developing. Since injected tumor cells distribute unevenly in the liver when the number of injected cells is relatively small, less than 0.5 × 10^6^ cells injection was not considered ideal to monitor using IVIS imaging. Moreover, since mice with 2.0x10^6^ cell injection showed rapid tumor growth and shorter survival compared to mice with 1.0x10^6^ cell injection, there might not be enough time to observe the efficacy or toxicity of study medications. Therefore, we considered that the 1.0x10^6^ cell injection model was the most suitable for the future preclinical experiments, and we next investigated the therapeutic effect of a signaling inhibitor by using this model. As shown in Fig. 7, the therapeutic effects of a MEK inhibitor, trametinib, on metastatic UM were clearly demonstrated by in vivo live imaging of human UM-bearing NSG mice. Despite the lack of potentially important elements including human immune system and human species-specific soluble factors such as hepatocyte growth factor (HGF), this splenic injection mouse model is suitable to quickly screen medications for the treatment of metastatic UM.

Grossniklaus’ group examined liver specimens from 15 UM patients with who had died of their disseminated disease. They found 2 distinct growth patterns of UM metastasis in the liver: infiltrative (n = 12) and nodular (n = 3) [35]. The infiltrative growth pattern showed cell growth within the sinusoidal space. The nodular growth pattern predominantly contained nodules of tumor that effaced, rather than infiltrated, the adjacent hepatocytes. During the development of orthotopic liver metastasis models of UM, we investigated the pattern of liver tumor development by direct tumor implantation to the liver as well as intra-splenic injection of tumor cells [22]. In the splenic injection model, small clusters of tumor cells reside in sinusoids at the early stage of metastasis and form microscopic tumor foci. Subsequently, the tumor cells form a larger cluster and infiltrate the hepatic parenchyma. Eventually the tumor cells occupy the hepatic lobule. The pattern of progression is similar to infiltrative pattern of metastasis in patients with metastatic UM and other types of cancer [35, 36]. Therefore, this splenic injection model is suitable for investigation on mechanism of systemic metastasis of uveal melanoma.

We previously have stained tumor tissue sections with several melanoma markers (S100, HMB45 or Melan-A) to pathologically confirm the presence of UM cells in orthotopic MUM mouse models [18, 22]. In our present study, we stained the liver, lung, and spleen of each mouse with SOX-10, which was recently reported to be the most sensitive marker for UM. SOX-10 was specifically expressed in tumors established by injection of UM001 cell and UM004 cell. Each tumor cell could be identified easily as a single cell because SOX-10 was stained in the nucleus. Regarding lung metastasis, no SOX-10-positive cells were observed in the lung at 6 weeks after splenic injection with splenectomy indicating that lung metastasis may be caused by systemic spread from the established hepatic tumors. It is of note that the tail vein injection did not result in lung metastasis. In this regard, settlement of much higher numbers of MUM cell in the liver by splenic or liver injection might have facilitated adaptation of MUM cells in the lung.

In order to discover clinically relevant treatments, laboratory tumor models must recapitulate the patient tumor as much as possible, whereby the treatment response in the patients can be predicted. In recent years, patient-derived xenograft (PDX) mouse models and organoid cultures have been used as tumor models close to human because they retain key characteristics of original tumors obtained from patients. Histological characteristics, genomic signatures, and heterogeneity of patient cancer cells tend to be maintained in these models. These models are considered to be clinically relevant to cancer models and represent a highly predictive drug response platform that resembles the therapeutic outcome in human patients [37–40]. However, these models are relatively expensive and have shown low success rates in establishment [39–41]. In this present study, we demonstrate that our orthotopic xenograft model by injecting MUM cells into the spleen establishes tumors relatively easily with low cost and consistency. Usage of IVIS live imaging for tumor progression assessment is more conventional and also reduces the numbers of mice required for individual experiments. Furthermore, this model is useful for investigation associated with the tumor microenvironment and the development of hepatic metastasis. In this regard, carefully characterized orthotopic xenograft models using established tumor cell lines are considered to be more suitable for rapid screening of drugs for potential clinical efficacy. Furthermore, we are developing a new mouse model in which MUM cells obtained from the PDX tumors will be injected to the spleen so that characteristics of MUM cells are closer to the parental MUM cells, compared to long-term cultured cell lines.

Despite above-mentioned benefits, this mouse model has some limitations. First, there is no human immune system in NSG mouse. Therefore, animal studies using NSG mice cannot assess the association between immune cells and tumor growth, and cannot be utilized for drug efficacy experiments including immunological reagents. The development of mouse models with human-like immune system or humanized immune microenvironment in tumors need to be explored. In this regard, syngeneic murine UM models would be more ideal to investigate the interactions between UM cells and the immune system although suitable syngeneic mouse UM models with hepatic tropism are not currently available. Second, we monitored hepatic tumor with IVIS in only one UM cell line in our present study. We demonstrated that splenic injection of another UM metastatic cell line, TJU-UM004, could establish hepatic tumors similar to UM001. Therefore, this model should be applicable to other types of tumor cell lines although optimization of individual tumor models is required. Additionally, we might monitor the growth of hepatic tumors without cell transfection by using PET-CT [42, 43] so that freshly isolated MUM cells could be tested for drug intervention. Third, tumors established from human UM cell lines might require supports from the surrounding normal hepatic tissue for their survival. For example, UM tumors developed in the liver of NSG mice contains mouse-derived blood vessels in the tumor [22]. However, some molecules secreted from the mouse liver, including growth factors, cytokines, and chemokines, may not work for humans due to species-specificity. In our previous study, we needed to use human HGF knock-in mice for drug resistance experiments related to MEK inhibitor because human HGF which was identified as a resistance factor for MEK inhibitors to human UM liver metastases was not present in standard NSG mice and mouse HGF didn’t interact with human c-Met [19, 20]. We need to investigate whether this splenic injection model can be applied to other types of mice such as HGF knock-in mice. Additionally, the growth of hepatic tumors established by the injection of MUM cells was much slower compared to liver metastasis mouse models using cutaneous melanoma [44, 45]. We also notice that our established MUM cell lines generally grow much slower than other cancer cell lines. Obviously, experiment conditions and tumor cell characteristics are completely different between cutaneous melanoma and UM [1, 14], and simple comparison would not be meaningful; however, the interaction between tumor cells and surrounding microenvironment might determine such difference and need to be further investigated.

## Conclusion

In conclusion, we established a new orthotopic hepatic metastatic xenograft mouse model by splenic injection of MUM cells. Moreover, the growth of orthotopic hepatic tumors, which were diffusely developed throughout the mouse liver, could be monitored with IVIS live imaging. Since orthotopic hepatic tumor via hematogenous dissemination can be established without complicated surgical technique, this model is expected to be widely applicable to the preclinical screening of multiple drug candidates for treatment of various types of cancers.

## Data Availability

Not applicable.

## Abbreviations

UM: Uveal melanoma
MUM: Metastatic uveal melanoma
HSCs: Hepatic stellate cells
FGF2: Fibroblast growth factor 2
NSG: NOD.Cg-Prkdc^scid^ Il2rg^tm1Wjl^/SzJ
IVIS: In vivo imaging system
SEMs: Standard errors of the means
